# Breast cancer patients’ presentation for oncological treatment: a single centre study

**DOI:** 10.11604/pamj.2016.24.63.8432

**Published:** 2016-05-13

**Authors:** Akinbolaji Andrew Akinkuolie, Amarachukwu Chiduziem Etonyeaku, Olalekan Olasehinde, Olukayode Adeolu Arowolo, Rereloluwa Nicodemus Babalola

**Affiliations:** 1 Department of Surgery, Obafemi Awolowo University, Ile-Ife, Nigeria; 2 Department of Surgery, Obafemi Awolowo University, Teaching Hospitals Complex Ile-Ife, Nigeria

**Keywords:** Breast cancer, presentation, oncological treatment

## Abstract

**Introduction:**

Breast cancer patients are presenting at advanced stages for oncological treatment in Nigeria and World Health Organization predicted developing countries’ breast cancer incidence and mortality to increase by year 2020.

**Methods:**

Prospective observational hospital based study that enrolled breast cancer patients from catchment area of an oncology service hospital in Nigeria between 2007 and 2013. Patients’ demographics, breast cancer burden and health care giver presentation variables were analysed for causal factors of seeking medical help and what determines commencement of effective oncological treatment.

**Results:**

Forty-six patients were enrolled, 19.6% of them presented primarily to oncologist while 80.4% presented secondarily for oncological treatment. There is a significant difference in presentation time for oncological treatment (t = -3.56, df = 42.90, p = 0.001) between primary (M =11.56 ± 5.21 weeks) and secondary presentation (M= 52.56 ± 10.27weeks). Tumor burden of those that presented secondarily were significantly more advanced (U = 78.5, p = 0.011) and, univariate analysis reveals that: *patients’ matrimonial setting, breast cancer awareness and mode of discovery of breast symptoms* are patient related factors that determines their choice of health care providers and, determinant of effective oncological treatment is patient first contact health care provider.

**Conclusion:**

Patients’ bio-characteristics that determine their choice of health care provider should be incorporated into community breast cancer sensitization drives. Additionally, there is a need for a government agency assign the task of accrediting and defining scope of enterprise of health care institutions and their health care providers in our pluralist health system.

## Introduction

Breast cancer is the commonest cancer among women worldwide and the most common cause of cancer related morbidity and mortality [1–3]. There is marked geographical variation in morbidity and mortality of breast cancer between the developed and developing countries [4–7]. Breast cancer screening and early presentation has changed the dismal outcome of this disease in the developed countries where despite increasing incidence, the morbidity and mortality are declining [4, 8]. However, the contrary is true in developing countries especially Nigeria where despite alarming increase in incidence, about 70 - 80% of breast cancer patients are still presenting with locally advanced or metastatic breast cancer [9–12]. More worrisome is the World Health Organization (WHO) report that expects world breast cancer incidence and mortality to increase by 50% by 2020 and this increase is anticipated to be highest in the developing countries of which Nigeria is inclusive: where 55% increase in incidence and 58% increase in mortality is predicted [13, 14]. In preparing for this anticipated increase, priority in developing countries should be to formulate breast cancer control strategies such as early breast cancer detection and early presentation for effective oncological treatment [15, 16]. The objective of this study is to find out patients’ related factors promoting early presentation and causal factor(s) of effective oncological treatment with the aim of reducing breast cancer related morbidity and mortality.

## Methods

This is a cross-sectional hospital based study carried out at Wesley Guild Hospital (WGH) in Ilesa, between January 2007 and December 2013. Ilesa is a cosmopolitan town situated in Osun States in south western Nigeria. The town has good roads network connection and transport system, it health system is pluralist made up of orthodox and modern health facilities and the modern health facilities consist of private and government owned hospitals. WGH is one of the governments owned hospital and is a satellite hospital of Obafemi Awolowo University Teaching Hospitals Complex (OAUTHC), Ile-Ife, Nigeria. Eligibility for the study is being a breast cancer patient presenting at the surgical oncology unit of WGH consenting to recruitment and data collected to be used for research purpose. Inclusion criteria were: being domicile in Ilesha and malignant breast lesion. Exclusion criteria were: being domicile outside Ilesha, benign breast lesion and inability to provide adequate information because of karnofsky performance status. Data obtained were patients’ demographics, clinical and laboratory details of breast cancer burden using American Joint Committee on Cancer (AJCC) staging system and evolution of patient's presentation to health care provider(s). Patients were subdivided into two groups whether they presenting primarily or secondarily for oncological treatment Data obtained were entered into the proforma designed for the study and analysed using Statistical Package for Social Sciences version 22. Descriptive statistics were employed for univariate analysis and, independent samples t test, nonparametric tests and regression analysis for multivariate analysis.

## Results

**Figure 1:**
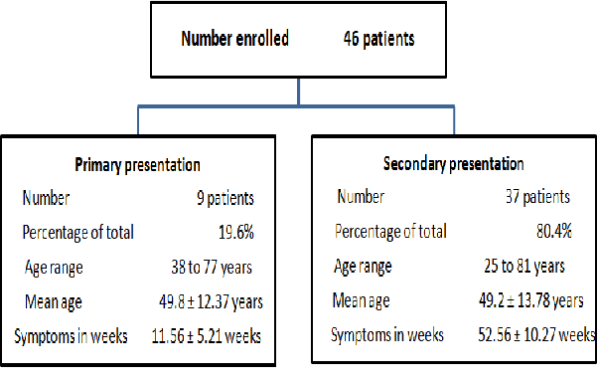
Summary of enrolled patients

**Figure 2:**
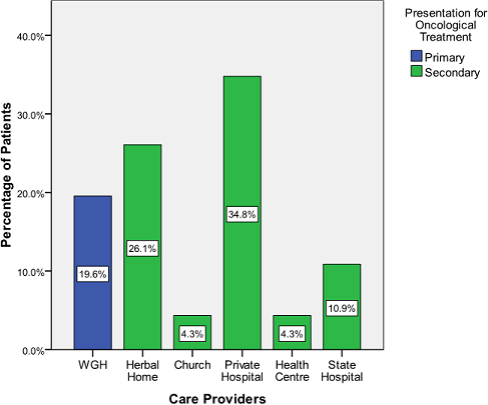
Patient's distribution at first health care provider's contact

**Figure 3:**
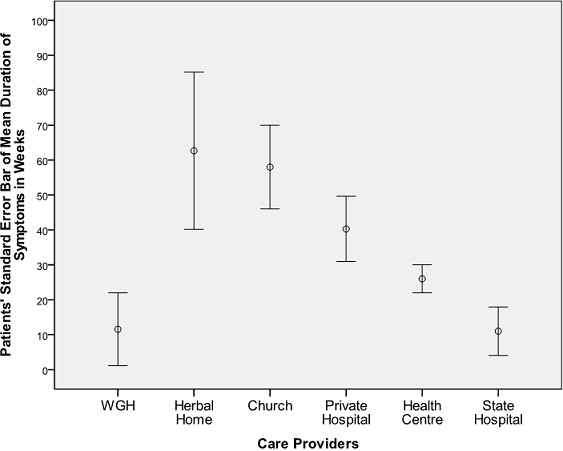
Patients’ first health care providers and mean duration of symptoms before oncological treatment

**Figure 4:**
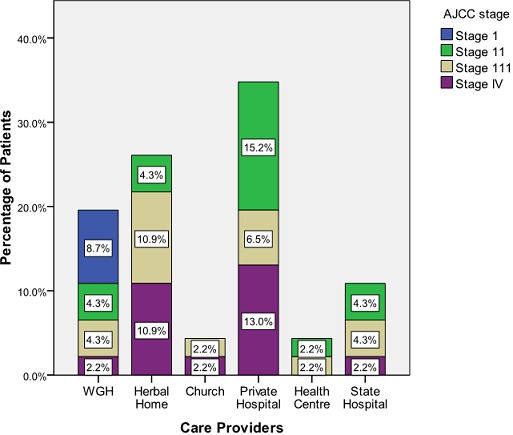
Distribution of patients’ AJCC staging at first oncological treatment

Forty-six patients met the inclusion criteria. They were all females; nine of these patients presented primarily in WGH while the remaining 37 presented secondarily for oncological treatment. Details of their demographic bio- characteristics are as shown in Figure 1 and Table 1. Patient first contact with health care provider distribution (Figure 2) reveals that 60.9% of the sum total of patients presented primarily either at herbal or private hospital. The mean presentation time in weeks for oncological treatment (Figure 3) was longest - 89 weeks - in patients from herbal home and the tumour burden was much advanced (Figure 4) in patients from herbal home and private hospitals. There is a significant difference in presentation time between those that presented primarily (M =11.56 ± 5.21 weeks) and those that presented secondarily (M = 52.56 ± 10.27weeks) for oncological treatment: ( t = -3.56, df = 42.90, two tailed p = 0.001). There is also significant difference in tumour burden between those that presented primarily and those that presented secondarily (U = 78.5, p = 0.011). Table 2 shows care providers’ documentation of patients’ disease., None of the patients from herbal home and mission house had any documentation of their disease and only about half of patients from private hospitals had documentation. In multivariate analysis significant patients’ related factors promoting early presentation to their choice of health care provider (Table 3) are: *their matrimonial setting, breast cancer awareness and mode of discovery of breast symptoms and, the significant determinant whether a breast cancer patient will have effective oncological treatment is their first healthcare provider* (Table 4).

**Table 1:** Patients’ demographic bio- characteristics

		Health care providers					
		WGH	Herbal home	Church	Private hospital	Health centre	State hospital
		N = 9	N = 12	N = 2	N = 16	N = 2	N = 5
Educational level	None	0	8	0	1	0	0
	Primary	4	2	2	4	0	1
	Secondary	1	1	0	5	0	2
	Tertiary	4	1	0	6	2	2
							
Monthly income	Low	8	9	2	6	0	1
	Medium	0	2	0	6	0	3
	High	1	1	0	4	2	1
							
Occupation	Unskilled	4	10	1	6	0	2
	Artisan	1	1	1	3	0	0
	Professional	4	1	0	7	2	3
							
Breast cancer awareness	No	5	11	2	13	1	3
	Yes	4	1	0	3	1	2
							
Matrimonial setting	Monogamy	4	4	2	14	2	5
	2 wives	4	3	0	2	0	0
	3 wives	1	2	0	0	0	0
	> 3 wives	0	3	0	0	0	0
							
Marital status	Single	0	0	0	0	0	0
	Married	5	8	1	12	1	4
	Divorced	3	0	1	1	1	0
	Widow	1	4	0	3	0	1
							
How discovered	Accidental	7	10	2	13	2	3
	Self breast examination	2	1	0	2	0	2
	Clinical breast examination	0	0	0	1	0	0
	Nipple discharge	0	0	0	0	0	0
	Breast discomfort	0	1	0	0	0	0
	Mammography	0	0	0	0	0	0

**Table 2:** Health care providers’ documentation of patients’ disease

	Health care providers						Total
	Primary	Secondary					
	WGH	Herbal home	Church	Private hospital	Health centre	State hospital	
Tissue diagnosis and/or referral note	9	0	0	5	0	1	15
Referral note only	0	0	0	0	2	4	6
No referral or tissue diagnosis	0	12	2	11	0	0	25
**Total**	9	12	2	16	2	5	46

**Table 3:** Determinants of patients’ early presentation to health care providers

Factors	P Value
Age	0.599
Educational level	0.479
Monthly income	0.098
Occupation	0.999
Breast cancer awareness	0.011
Matrimonial setting	0.010
Marital Status	0.967
How discovered	0.023

Multinominal regression P < 0.05

**Table 4:** Determinants of effective oncological treatment

Factors	P Value.
Age	0.228
Educational level	0.089
Monthly income	0.128
Occupation	0.491
Breast cancer awareness	0.649
Matrimonial setting	0.796
Marital Status	0.925
How discovered	0.951
Health care providers at first contact	0.001
Health care providers at secondary contact	0.413

Linear regression *P < 0.05*

## Discussion

Presentation of breast cancer patients at advanced stage is a major contributor to increasing morbidity and mortality in the developing countries [14, 17]. Reviews from other developing countries (Table 5) highlight reasons for advanced stage presentation from various nations. The variations of causes from nation to nation may be related to their socio-cultural background [18, 19]; therefore, it is important to know the genesis of advanced stage presentation in our environment so as to incorporate this into future breast cancer campaign. Outcome of this study reveals that 19.6% of the patients presented directly in an institution where they had access to oncological treatment while the remaining 80.4% presented primarily at centres with limited resources for breast cancer management with significant difference in the disease burden and presentation time in the two categories of patients. Though, matrimonial setting, breast cancer awareness and mode of discovery of breast symptoms are prominent patients’ related factors promoting early presentation to their first health care provider; the relative importance these factors may be complex and interrelated. The role of matrimonial setting in the choice of health care provider may be related to family belief about breast cancer [20], treaty with a family doctor/spiritualist, and previous family experience of poor outcome of close relatives with breast cancer treated at modern health facility [21]. Though, it is commonly argued that the final health related decision rested with the patient but in African context it is a taboo for a woman to disobey her spouse or close relative even at the risk of her health. Therefore, campaigns directed at early breast cancer presentation should not only be aimed at patients but also at her family. Breast cancer awareness and mode of discovery of breast symptoms as determinants of health care provider presentation reflect patient knowledge of the disease and the disease outcome [22]. Surprisingly, breast cancer patient's level of education is not a determinant of her early presentation to a health care provider in this study (Table 3); probably academic certification may not be synonymous with awareness of a disease and where to obtain appropriate treatment [23]. Thus literacy may not necessarily infer knowledge of a disease and this should be considered during breast cancer sensitization drives.

**Table 5:** Reviews of presentation of breast cancer patients from developing countries

Authors	Region	Reason for advanced stage presentation
Poum, A., et al. (2013)	Thailand	Higher family income Smoking Delayed referral by health care providers
Ghazali, S. M., et al. (2013	Malaysia	Non-practice of breast self examination Marital status
Alhurishi, S., et al. (2011	Middle East	Older age Lower educational level Lack of family of history of breast cancer
Akhtar, M., et al. (2011).	India	Lack of awareness of breast cancer Financial constraints Non referral by general practitioners
Otieno, E. S., et al. (2010).	Kenya	Fear of diagnosis Painlessness of breast pathology False assurance by health providers
Clegg-Lamptey, J., et al. (2009)	Ghana	Previous medical consultations Ignorance Fear of mastectomy Herbal treatment Prayer/prayer camps Financial incapability

The result of this study shows the dominant role of a health care provider at first patients’ contact as determinant of effective oncological treatment, though being tag qualified health care personnel is at the prerogative of the government who is the licensing body; unfortunately, 80.4% of the patients presented outside the setting where they can access oncological management. Health care providers’ documentation of patients’ disease in Table 2 may be from misdiagnosis [24], lack of knowledge of the disease and available treatment options [25]. Additionally, it may be from poor judgement about the need for referral to the appropriate health care provider [26, 27]. Improvement in breast cancer outcome in our society can still be accomplished within the existing pluralist health care system through practical interventions that are realistic and efficient [17, 21]. The goal of future research will be to find out what implementation strategies can most effectively guide our pluralist health system reorganization to improve breast cancer outcome. In this regard, there is need for evaluation of appropriate certification and re -accreditation of health care institutions and their permissible scope of enterprise on breast cancer outcome and the effect of disease recognition training in oncology for health care providers operating herbal and convalescence homes on breast cancer presentation. Additionally, there is urgent need for upgrading existing government health care infrastructures and training of personnel in oncology to cope with oncological challenges of years to come.

## Conclusion

Presentation of breast cancer patients outside the setting where they can access early and effective oncological treatment seems to forms the basis of advanced stage presentation. *Matrimonial setting, breast cancer awareness and mode of discovery of breast symptoms* are significant patients’ bio-characteristics that determine their choice of health care provider. These factors should be considered in community breast cancer sensitization drives. Additionally, non oncologist health care providers in our pluralist health system will need re-training in oncology and their scope of enterprise should be properly defined by appropriate government institution.

### What is known about this topic

- Seventy to eighty percent of breast cancer patients in Nigeria present primarily at oncological centre for treatment with locally advanced or metastatic disease;
- Breast cancer awareness is the determinant of early presentation to a health care provider;
- Determinant of effective oncological treatment is patient presentation for treatment.

### What this study adds

- Only about twenty percent of breast cancer patients in this study present primarily at oncological centre for treatment and about sixty percent of them presented with early breast cancer;
- Breast cancer awareness, patient's matrimonial setting and mode of discovery of breast symptom are the determinants of early presentation to a health care provider;
- Determinant of effective oncological treatment is the health care provider at first patient/health care provider contact.
